# Selection of *Salmonella enterica* Serovar Typhi Genes Involved during Interaction with Human Macrophages by Screening of a Transposon Mutant Library

**DOI:** 10.1371/journal.pone.0036643

**Published:** 2012-05-04

**Authors:** Sébastien C. Sabbagh, Christine Lepage, Michael McClelland, France Daigle

**Affiliations:** 1 Department of Microbiology and Immunology, University of Montreal, Montreal, Quebec, Canada; 2 Vaccine Research Institute of San Diego, San Diego, California, United States of America; 3 Department of Pathology and Laboratory Medicine, School of Medicine, University of California Irvine, Irvine, California, United States of America; University of Osnabrueck, Germany

## Abstract

The human-adapted *Salmonella enterica* serovar Typhi (*S*. Typhi) causes a systemic infection known as typhoid fever. This disease relies on the ability of the bacterium to survive within macrophages. In order to identify genes involved during interaction with macrophages, a pool of approximately 10^5^ transposon mutants of *S*. Typhi was subjected to three serial passages of 24 hours through human macrophages. Mutants recovered from infected macrophages (output) were compared to the initial pool (input) and those significantly underrepresented resulted in the identification of 130 genes encoding for cell membrane components, fimbriae, flagella, regulatory processes, pathogenesis, and many genes of unknown function. Defined deletions in 28 genes or gene clusters were created and mutants were evaluated in competitive and individual infection assays for uptake and intracellular survival during interaction with human macrophages. Overall, 26 mutants had defects in the competitive assay and 14 mutants had defects in the individual assay. Twelve mutants had defects in both assays, including *acrA*, *exbDB*, *flhCD*, *fliC*, *gppA*, *mlc*, *pgtE*, *typA*, *waaQGP*, SPI-4, *STY1867-68*, and *STY2346*. The complementation of several mutants by expression of plasmid-borne wild-type genes or gene clusters reversed defects, confirming that the phenotypic impairments within macrophages were gene-specific. In this study, 35 novel phenotypes of either uptake or intracellular survival in macrophages were associated with *Salmonella* genes. Moreover, these results reveal several genes encoding molecular mechanisms not previously known to be involved in systemic infection by human-adapted typhoidal *Salmonella* that will need to be elucidated.

## Introduction

The human specific pathogenic bacteria *Salmonella enterica* serovar Typhi (*S*. Typhi) is responsible for the systemic infection known as typhoid fever. Epidemiological studies show that in 2000, typhoid fever caused nearly 22 million infections, while killing more than 200 000 individuals in endemic countries of the world [1]. Unfortunately, multidrug-resistant strains of *S*. Typhi are arising in many of these regions, rendering typhoid treatment more complex and difficult than ever [2]. Hence, there is a critical need to uncover new potential therapeutic targets within this pathogen, leading to either new antimicrobials or more effective vaccine therapy against typhoid fever [3].

Survival of *Salmonella* within macrophages is crucial for systemic infection, since mutants that fail to replicate in these cells *in vitro* are avirulent in animals [4]. Thus, macrophage infection represents a good model for the study of *Salmonella* genes involved in virulence. Genes playing a role in intracellular survival within macrophages have been identified. Some of these include the *phoPQ* regulatory system [5]–[8] as well as components of the type three secretion system (T3SS) encoded by *Salmonella* pathogenicity island (SPI)-2 genes [9]–[11]. The critical role of SPI-2 T3SS regarding intramacrophage survival has been clearly demonstrated for *Salmonella enterica* serovar Typhimurium (*S*. Typhimurium) [11]–[13], which causes localized gastroenteritis during human infection and systemic disease in mice. Interestingly, although both *S*. Typhi and *S*. Typhimurium serovars are closely related, and have about 90% of their genomes in common [14], SPI-2 T3SS was not essential for *S*. Typhi survival within human macrophages [8], whereas no major differences in SPI-2 T3SS genetic composition were observed between both serovars [15]. Vaccine development has also witnessed many situations where inactivation of the same genes in both serovars led to different phenotypes in the animal model or human host [16]–[21]. Moreover, the intracellular strategies used by *Salmonella* are not fully understood, suggesting that several genes used by the bacteria to survive inside macrophages remain to be identified.

Screening methods have been developed to comprehensively identify genes involved during infection. Signature-tagged mutagenesis (STM) was the first technique to simultaneously screen a pool of mutants obtained by transposon insertion to identify virulence genes in an animal model [22]. With this strategy, mutants from the initial input pool that are underrepresented in the output pool point towards candidate genes potentially involved during infection. Recent advances, including genome sequencing and microarrays, have led to even more sophisticated screening techniques and have been used successfully to identify virulence genes of *S*. Typhimurium in macrophages and within mice [23]–[26]. In order to extend our knowledge on the genetic determinants of *Salmonella* used during interaction with macrophages, such a strategy was applied to identify genes for which mutants were underrepresented following competitive passage of a transposon mutant library through macrophages, with the unprecedented use of serovar Typhi and human macrophages. Following the screening, isogenic markerless deletion mutants were created to verify the phenotypes associated with uptake and intracellular survival, using mixed and individual infection experiments. Most importantly, several new *S*. Typhi genes involved during interaction with human macrophages were identified.

## Materials and Methods

### Bacterial strains and plasmids

Strains and plasmids used in this study are listed in Table S1. Bacteria were routinely grown overnight statically (low aeration) in Luria-Bertani (LB) broth and plates at 37°C, unless indicated. Auxotrophy among deletion mutants was tested using M63-glucose minimal medium supplemented with 40 mg/L tryptophan, 40 mg/L cysteine, and 0.6% (w/v) glucose during overnight growth and optical densities at 600 nm (OD_600_) of these cultures were compared to that of the wild-type. In order to evaluate resistance of mutants to the detergent sodium deoxycholate (DOC), bacteria were grown overnight in RPMI 1640 (Wisent) medium containing 0.1% (w/v) DOC. OD_600_ were compared to that of the wild-type. To test survival of mutant *acrA* in DOC, 5×10^6^ CFUs of an overnight culture grown statically were inoculated in 1 ml of 0.1% (w/v) DOC in PBS. Samples were left on ice and viable bacteria were determined as CFUs at 0 and 2 hours (h) after inoculation. When necessary, antibiotics or supplements were added at concentrations of 50 µg ml^−1^ for ampicillin (Ap), kanamycin (Km), nalidixic acid (Nal) or diaminopimelic acid (DAP); 34 µg ml^−1^ for chloramphenicol (Cm); or 50 µM isopropyl-β-D-thiogalactopyranoside (IPTG). Transformation of bacterial strains was routinely done by using the calcium/manganese-based method or by electroporation [27].

### Construction of the transposon harbouring a T7 RNA polymerase promoter for generation of the mutant library

A PCR-based strategy was used to insert a T7 RNA polymerase promoter at the 3′ region of the mini-Tn*10*-Km transposon from the pLOFKm suicide conjugative plasmid [28], kindly provided by Kenneth E. Sanderson, University of Calgary. This vector contains a mini-Tn*10*-Km transposon with IS*10* inverted repeated sequences flanking a kanamycin resistance (Km^r^) cassette and an IPTG-inducible transposase located outside the mobile element. The Km^r^ cassette was amplified with a primer that added the T7 promoter at its 3′ end (Front NotI-Kan and Rear NotI-Kan) (Table S2). The PCR product was ligated into pLOFKm, both digested with *Not*I, thus creating pLOFKm-T7 (pSIF117). This plasmid was transformed into *Escherichia coli* (*E. coli*) MGN-617 [29] and conjugated into *S*. Typhi wild-type strain ISP1820 [30] with IPTG present in the agar mating plate. Approximately 10^5^ transposon insertion mutants (Km^r^) were obtained and represent the library of mutants used for our screening.

### Competitive selection of the mutant library in cultured macrophages

The human monocyte cell line THP-1 (ATCC TIB-202) was maintained in RPMI 1640 containing 10% (v/v) heat-inactivated fetal bovine serum (Wisent), 25 mM HEPES (Wisent), 2 mM L-glutamine (Wisent), 1 mM sodium pyruvate (Wisent) and 1% modified Eagle's medium nonessential amino acids (Wisent). A stock culture of these cells was maintained as monocyte-like, non-adherent cells at 37°C in an atmosphere containing 5% (v/v) CO_2_. For screening, 10^7^ macrophages were seeded in a 100×20 mm Petri dish (Sarstedt) and differentiated by addition of 10^−7^ M phorbol 12-myristate 13-acetate for 48 h. Prior to infection, the macrophage supernatant was changed with fresh medium at 37°C. The mutant library (used as input pool) was grown overnight statically in 20 ml LB with Km at 37°C and used to infect two separate macrophage monolayers (generating two independent output pools). Bacteria were added at a multiplicity of infection (MOI) of 10 to the cell monolayer and incubated at 37°C for 30 minutes to allow internalization. This corresponds to the initial interaction with cells, where some bacteria will be associated (adherence) and some will be intracellular (uptake). Cells were then washed three times with prewarmed PBS, pH 7.4, and medium containing 100 µg ml^−1^ of gentamicin (Wisent) was added to kill extracellular bacteria (0 h). After 2 h of incubation with high-concentration gentamicin at 37°C, cells were washed and medium containing 12 µg ml^−1^ of gentamicin was added for the remainder of the experiment. After 22 h of low-concentration gentamicin treatment (24 h post-infection), cells were washed and lysed by addition of 10 ml 0.1% (w/v) DOC in PBS. The lysate was centrifuged and bacteria were resuspended in 20 ml LB with Km and grown overnight statically at 37°C for the next serial passage in macrophages. After three serial passages through macrophages, bacteria released from infected macrophages were grown overnight with agitation in LB with Km at 37°C and represent the output pools (two output pools were generated in parallel). Bacterial cultures from the macrophage output pools and from the initial input pool were used for genomic DNA extraction.

### Amplification and labelling of transposon-flanking sequences

Genomic DNA from the input and output pools was isolated using phenol/chloroform extraction, followed by ethanol precipitation [31]. Four µg of genomic DNA from each pool was sonicated using five pulses of two seconds each, with a Sonics & Materials Vibra-cell VC600 device (Sonics & Materials Inc., Danbury, CT). Sonicated genomic DNA was then poly(A)-tailed with terminal transferase (TdT) (New England Biolabs) and purified as described previously [23]. Purified sonicated poly(A)-tailed DNA obtained from input and output pools was used as template for nested PCR reactions as previously described [23] with some modifications, in order to specifically amplify the segments encompassing the 3′ end of the transposon (including the inserted T7 RNA polymerase promoter) and the adjacent genomic DNA region. Briefly, 50 ng of purified sonicated poly(A)-tailed DNA was included in the first round of nested PCR in a total volume of 25 µL. This reaction combined 1× PCR buffer, 0.2 mM dNTPs, 2.5 mM MgCl_2_, 0.2 µM primers pLOF F seq and CCT_24_VN (the latter made to anneal to the poly(A)-tail), and 1.25 U Taq polymerase (Feldan). The PCR steps followed were: initial denaturation (hot start) at 94°C for 1 minute, then 30 cycles including denaturation at 94°C for 30 seconds, annealing at 50°C for 30 seconds, and elongation at 72°C for 30 seconds. The reaction ended with one last elongation at 72°C for 3 minutes. The second round of nested PCR was done in a volume of 50 µL and included 1 µL of the first PCR amplification reaction, internal primer STY:PCRNiche#2 and primer CCT_24_VN used in the first round. The amplified DNA was then subjected to an *in vitro* transcription reaction, using the MEGAscript T7 High yield transcription kit (Ambion) with 5 µL of the nested PCR reaction included directly as the template in a 20 µL reaction, by following the manufacturer's protocol with some modifications. Briefly, the transcription reaction was done at 37°C for 2 h, and subsequently, the synthesized RNA was treated with DNase (Ambion) for 30 minutes at 37°C, purified with the RNeasy Mini kit (Qiagen) and eluted in RNase-free H_2_O.

Purified RNA was used to synthesize labelled cDNA probes as described previously [32], except that 4.8 µg of total RNA were added to 4 µg of random hexamers (Sigma) and reverse transcribed using SuperScript II reverse transcriptase (Invitrogen) while incorporating Cy5-dCTP (Amersham Biosciences) for the input (control sample) and Cy3-dCTP (Amersham Biosciences) for the output (experimental sample). Labelled first-strand cDNA was column-purified using QIAquick PCR purification kit (Qiagen), and eluted with RNase-free H_2_O.

### Microarray hybridization of labelled cDNA

The non-redundant *Salmonella* microarray that comprises >98% of *S*. Typhi strain CT18 genes was used as described previously [32], [33]. Microarrays were scanned using a GenePix 4000B laser scanner (Molecular Devices) at 5 µm resolution and signal intensities were quantified with GenePix Pro 6.0 (Axon Instruments). Background subtraction was done with GenePix Pro, by applying the software's default «local median intensity» subtraction method. Results were normalized and analyzed using WebArrayDB (http://www.webarraydb.org) [34]. Genes with a signal intensity two standard deviations above the average background level were considered as detected [32]. The array platform and hybridization data are MIAME-compliantly deposited at http://www.webarraydb.org (MPMDB ID 144).

### Generation of individual mutants and complementation

Gene deletions were generated by allelic exchange as described previously [30], by using the overlap-extension PCR method [35]. Primers used for each gene are listed in Table S2. Mutations were confirmed by PCR. Complementation of mutants was performed by cloning an intact copy of the *S*. Typhi wild-type gene or gene cluster into the low-copy-number vector pWSK29 [36]. This plasmid has been shown to have no deleterious effect on *S*. Typhi infection of host cells [37]. For infection assays, the complemented strains were grown overnight in LB with Ap.

### Infection assays of cultured human macrophages

For competitive index (CI) experiments in macrophages [38], a spontaneous nalidixic acid-resistant (Nal^r^) *S*. Typhi ISP1820 strain was used as the wild-type (DEF566). This Nal^r^ strain showed no intracellular attenuation compared with strain ISP1820 when both were used in a CI experiment in THP-1 macrophages [8]. The wild-type and mutant strains used for competition experiments were separately grown overnight (static) in LB broth and the concentration (CFU/ml) of each strain was evaluated by OD_600_ of the suspension culture. CFU counts obtained by plating on LB agar with and without antibiotic assessed that the mixture contained equivalent numbers of actual viable bacteria from each strain. The 1∶1 mixture (CFU/ml) of the two cultures was added to the THP-1 cell monolayer as described above for screening of the mutant library, except that cells were seeded at 5×10^5^ cells per well in 24-well tissue-culture dishes at an MOI of 50 [38]. Cells were lysed by addition of 1 ml 0.1% (w/v) DOC in PBS per well, and the numbers of viable intracellular bacteria were determined as CFUs at 0 and 24 h after infection by plating on LB agar and on LB agar with antibiotic. For CI experiments involving plasmid-rescued mutant strains, the complemented mutant was grown overnight separately in LB broth with Ap, resuspended in LB without antibiotic, and used to prepare a 1∶1 mixture (CFU/ml) with the mutant. The CI for bacterial uptake is defined as the mutant to wild-type ratio of bacteria recovered at 0 h divided by the equivalent ratio of bacteria in the mixed inoculum. The CI for bacterial survival is defined as the mutant to wild-type ratio of bacteria recovered at 24 h post-infection divided by the equivalent ratio of bacteria recovered at 0 h. Results are expressed as the mean ± standard error of the mean (SEM) of at least three independent experiments performed in duplicate and Student's two-tailed *t*-test was used for statistical analysis.

Macrophage infection with individual mutants was performed as described above for the CI assay, except that an MOI of 10 was used. Bacterial uptake was defined as the number of bacteria recovered at 0 h after infection divided by the number of bacteria in the inoculum. Survival was defined as the number of bacteria recovered 24 h after infection divided by the number of bacteria recovered at 0 h. In order to compare data from different experiments, the values representing recovery percentages were then normalized relative to that of the wild-type control, which was designated 100% at each time point, unless indicated. Results are expressed as the mean ± SEM of at least three independent experiments performed in duplicate and Student's two-tailed *t*-test was used for statistical analysis.

### Motility assay

Mutants were tested for their ability to swim in LB 0.3% agar plates as previously described [39], with some modifications. Prior to inoculation, the plates were allowed to dry for 1 h under a sterile laminar flow hood at room temperature. Strains were grown overnight with agitation in LB at 37°C, were then diluted 1∶100 in LB and grown with agitation at 37°C to an OD_600_ ranging from 0.4 to 0.5. Each mutant strain was tested along with the wild-type counterpart, where both were spotted into the agar using 6 µL of bacterial culture on the same swimming plate. The plates were incubated at 30°C for 16 to 17 h, except for mutant *typA*, whose swimming plate was incubated at 37°C for 10 to 11 h (this mutant showed a reduced growth rate at 30°C compared to 37°C; data not shown) and *typA*, also known as *bipA*, has been shown to be involved in growth at low temperature [40]–[43]. Following incubation, outward migration diameters for each mutant were measured and compared to that of the respective wild-type counterpart found on the same plate, in order to identify mutants exhibiting a swimming defect. Motility levels of mutants are expressed as the percentage obtained by dividing the mutant swimming diameter by that of the wild-type (Table 1). Results represent the mean ± SEM of at least three independent experimental replicates and Student's two-tailed *t*-test was used for statistical analysis.

**Table 1:** Summary of *S*. Typhi deletion mutants.

			Uptake/Survival defects observed in macrophages			
ORF(s) (gene name)^a^	Description	Fold-change^b^	*S*. Typhi^c^ (typhoid patients)^d^	Other *Salmonella* serovars	Motility level (%)^e^	H_2_O_2_ sensitivity (mm)^f^
**Cell envelope**						
*STY0520* (*acrA*)	Acriflavine resistance protein	−4.13	uptake/survival (AcrA)	uptake [63]	-	-
*STY2167* (*fliC*)	flagellin	−2.72	uptake/survival	uptake [97]	20 (±0.7)^g^	-
*STY2632* (*pgtE*)	outer membrane protease E	−3.20	uptake	survival [98]	-	-
*STY4071-73* (*waaQ* ***G*** *P*)	LPS core biosynthesis proteins	−3.25	uptake/survival	survival [92], [93]	62 (±11.9)^g^	3 (±0.6)
*STY2303-04* (*rfb* ***IC***)	O-Ag biosynthesis	−2.87/−3.48	survival	ND^h^	70 (±5.7)^g^	-
*STY0024-34* (*bcfABC* i *DE* ***F*** *GH*)	fimbrial structure	−2.12	uptake (BcfD)	ND	-	1.3 (±0.3)^g^
*STY1176-82* (*csgG* ***F*** *EDBAC*)	fimbrial structure	−4.74	uptake (CsgEFG)	ND	-	-
*STY0369-73* (*stbA* ***B*** *CDE*)	fimbrial structure	−3.69	uptake (StbD)	ND	-	-
*STY2378-81* (*stcABC* ***D***)	fimbrial structure	−2.85	survival	ND	-	2 (±1.0)
*STY0041*	putative exported protein	−5.75	survival	ND	-	-
*STY1358-67* j (**64**)	genetic island	−3.47	ND (*STY1364*)	ND	-	2.5 (±0.5)^g^
***STY1867-1868***	putative proteins	−5.46/−4.14	uptake/survival	ND	-	1.5 (±0.5)
**Pathogenesis**						
*STY2753-63a* (***sinH*** i)	pathogenicity island (CS54)	−2.32	uptake (ShdA^i^)	ND	-	-
*STY1878* (*pagC*)	outer membrane invasion protein	−2.23	uptake/survival (PagC)	survival [6]	-	-
*STY3004* (*sipF*)	acyl carrier protein (SPI-1)	−5.00	uptake/survival	ND	-	-
*STY4452-60* (*siiA* ***BC*** *D* ***E*** i *F*)	T1SS and adhesin (SPI-4)	−3.55/−3.43/−2.35	uptake/survival	ND	-	2 (±0.0)^g^
*STY4679* j	putative membrane protein (SPI-7)	−3.33	uptake	ND	84 (±2.6)	-
j *STY* ***4842*** *-43*	putative regulatory proteins (SPI-10)	−3.74	uptake/survival	ND	-	2.5 (±0.5)
**Regulatory functions**						
*STY2133-34* (*flhC* ***D***)	flagellar master regulators	−3.22	uptake/survival	ND	21 (±0.9)^g^	-
*STY3641* (*gppA*)	guanosine pentaphosphatase	−4.16	uptake/survival	ND	-	2 (±0.6)^g^
*STY1576* (*mlc*)	putative regulatory protein	−2.25	survival	ND	-	3 (±0.0)^g^
*STY3871* (*typA*)	GTP-binding protein	−2.94	uptake/survival	ND	76 (±4.5)^g^^, ^ k	2 (±0.0)
**Transport and binding proteins**						
*STY3331-32* (*exb* ***DB***)	biopolymer transport proteins	−6.04/−3.53	uptake/survival	ND	-	1.5 (±0.9)
*STY1649* (*ompN*)	outer membrane protein N	−3.87	survival	ND	-	-
**Unknown function**						
*STY0016*	hypothetical protein	−3.13	ND	ND	-	-
*STY1398*	hypothetical protein	−3.84	survival	ND	-	1.5 (±0.5)
*STY1869*	hypothetical protein	−2.10	uptake/survival	ND	-	-
*STY2346*	hypothetical protein	−4.47	uptake	ND	-	2 (±1.0)

a Loci which have been inactivated in each of the 28 markerless deletion mutants created for this study are listed. Numbers or characters in bold among a gene cluster represent ORF(s) or genes selected following initial screening through macrophages.

b Log_2_ of output/input values for genes identified following screening of mutant pool through macrophages. For gene clusters, the fold-changes are associated to selected genes (bold) among the cluster.

c Phenotypes have been deduced from results of competitive assays in combination with those from individual infections performed in this study using *S*. Typhi deletion mutants, the wild-type strain, and the nalidixic acid-resistant wild-type strain (DEF566).

d Gene or ORF products which are among *S*. Typhi antigens detected in blood of typhoid fever patients according to previous studies [56]–[58].

e Percentages represent mutant swimming diameter in mm divided by that of the wild-type, thus indicating the level of motility remaining for the mutants in comparison to the wild-type. Results represent the mean ± standard error of the mean (SEM) of at least three independent experimental replicates and Student's two-tailed *t*-test was used for statistical analysis.

f Values were obtained by subtracting mutant inhibition diameter in mm by that of the wild-type. Results represent the mean ± SEM of at least two independent experimental replicates and Student's two-tailed *t*-test was used for statistical analysis.

g Results for mutant are significantly different from those of the wild-type (*P*<0.05).

h ND, no defects during interaction with macrophages are reported in previously published literature on *Salmonella* serovars other than Typhi.

i Pseudogene in *S*. Typhi strains CT18 [66] and Ty2 [99].

j Unique to *S*. Typhi compared to *S*. Typhimurium [66].

k Motility assay was done at 37°C for Δ*typA*, instead of 30°C used for other mutants, since this mutant exhibited reduced *in vitro* growth at 30°C (data not shown).

### Sensitivity of mutant strains to hydrogen peroxide

Sensitivity to hydrogen peroxide (H_2_O_2_) was evaluated by an agar overlay diffusion method as previously described [44], with some modifications. Mutant and wild-type strains were grown overnight statically in LB at 37°C to an OD_600_ ranging from 0.5 to 0.6. For each strain, 100 µL of bacterial culture were mixed to 3 ml of molten top agar (0.5% agar). The mix was then poured evenly over an LB plate (1.5% agar) and let to dry at room temperature until the top agar had completely solidified. One filter paper disc (6 mm diameter; Becton Dickinson) was then placed at the center of the solidified overlay, and 10 µL of 29.9% H_2_O_2_ (Sigma) were spotted onto the disc. Plates were incubated overnight at 37°C, and following growth, the diameters of inhibition zones of the mutants were measured and compared to that of the wild-type counterpart. H_2_O_2_ sensitivity of mutants was defined as the difference in mm between the mutant inhibition zone and that of the wild-type (Table 1). Results represent the mean ± SEM of at least two independent experimental replicates and Student's two-tailed *t*-test was used for statistical analysis.

## Results

### Genome-wide mutagenesis of *S*. Typhi

In order to identify *S*. Typhi genes involved in interaction with human macrophages, a library of transposon insertion mutants was constructed by conjugative transfer of the mini-Tn*10*-T7 transposon into the *S*. Typhi wild-type strain ISP1820 [45]. A library of approximately 10^5^ mutants was generated. Southern blot analysis of some of the transposon mutants was used to verify and to confirm that the mutagenesis resulted in single random insertions (data not shown). Moreover, 3859 out of 4452 *S*. Typhi genes printed on the microarray were detected in the input library (data not shown).

### Competitive selection of transposon mutant library in macrophages

**Figure 1:**
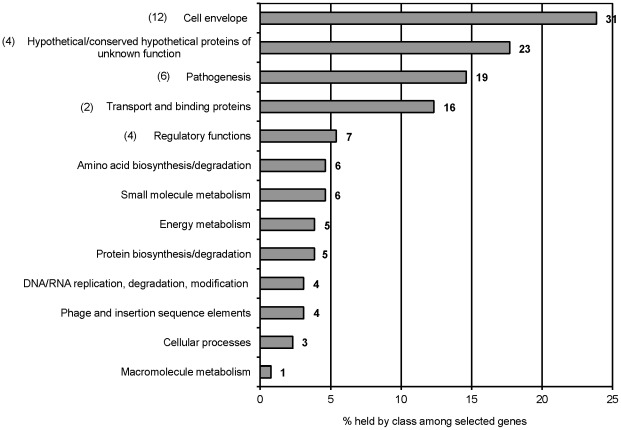
Functional classification of 130 *S*. Typhi genes identified following competitive selection in macrophages. Functional classes are indicated on the left and the number of genes within a class is indicated in bold on the right of each bar. The number of mutants created among each class is shown in parentheses on the left of each class.

The transposon mutant library was subjected to three rounds of competitive serial passages through human cultured THP-1 macrophages. Bacterial genomic DNA from output pools following passages through cells and from the unpassaged input pool was extracted. From these, probes corresponding to DNA adjacent to the transposon were synthesized and labelled for microarray hybridization, in order to target *S*. Typhi genes selected following competitive passages through macrophages. 130 genes were identified as potentially involved due to negative selection of transposon mutants (Table S3), by using a four-fold change threshold (input∶output ratio of 4∶1, 
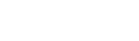
) and a *P*-value<0.0005. Among these selected genes, we found many for biogenesis of lipopolysaccharides (LPS), fimbriae and flagella, as well as virulence genes (Table S3). Additionally, many of these selected genes are part of SPIs-1, -2, -4, -5, -6, -7, -10, -11, -12, -13, and -16 from *S*. Typhi. The 130 genes, corresponding to mutants underrepresented during the competitive screening assay, were grouped into functional classes based mainly on the Sanger Institute classification (http://www.sanger.ac.uk) (Figure 1). The most highly represented classes were *Cell envelope* (24%), *Unknown function* (18%), *Pathogenesis* (15%), and *Transport and binding proteins* (12%). Only 10 of these 130 genes were previously selected as less fit during repeated passages of an *S*. Typhi mutant library in rich media (*btuC*, *gpmA*, *kdgA*, *mlc*, *prc*, *proC*, *rplS*, *ycdC*, *waaG*, and *ybiS*) (Table S3) [46].

### *In vitro* characterization of markerless deletion mutants

A total of 28 isogenic markerless deletion mutants were generated in *S*. Typhi strain ISP1820 (Table 1), chosen from the top functional classes of genes (Figure 1). The deletions were gene-specific and nonpolar or sometimes targeted a gene cluster, such as a complete operon (or putative operon) or a portion of it. Clusters include *exb* ***DB***, *waaQ* ***G*** *P*, *rfb* ***IC***, the *bcf*, *csg*, *stb*, and *stc* fimbrial operons, *STY1358-67*, *STY* ***1867-1868***, the CS54 pathogenicity island, SPI-4, *STY* ***4842*** *-4843* from SPI-10, and *flhC* ***D***. Each of these clusters includes at least one of the selected genes (bold) (Table S3). We first determined if gene inactivation of our mutants affected growth *in vitro*. All deletion mutants had growth curves in LB broth similar to that of the wild-type strain, except mutants *waaQGP* and *mlc* which had a slight defect (data not shown). Growth deficiency for these mutants has been previously observed [47], [48]. None of the mutants demonstrated auxotrophy when grown in M63-glucose minimal medium (data not shown). Resistance to the detergent DOC, as used in the infection assays, was also verified for all of the mutants. Only mutant *acrA* failed to grow overnight in RPMI containing 0.1% DOC and showed approximately 10% mortality over 2 h in PBS with 0,1% DOC. Motility is a property that has been previously associated with *S*. Typhi virulence towards eukaryotic cells [49], thus the abilities of the mutants to swim in soft agar was evaluated. Mutants *fliC*, *waaQGP*, *rfbIC*, *flhCD*, and *typA* swam significantly less in LB 0.3% agar plates than the wild-type strain (*P*<0.05), and mutant *STY4679* also exhibited a swimming defect that was not significant (*P* = 0.226) (Table 1). The motility defect was expected for mutants *flhCD* and *fliC*, as they represent the master regulators and main structural subunit of the flagellar system, respectively [50]. Mutation of *waaG* (*rfaG*) was previously shown to affect swimming motility in *E. coli*, *S*. Typhimurium and *S*. Typhi [47], [51], [52]. Phagocytic cells are able to produce reactive oxygen species, such as H_2_O_2_, as part of defense mechanisms against invading microorganisms [53]. Hence, we investigated sensitivity of the mutants when exposed to oxidative stress mediated by H_2_O_2_. Mutants *waaQGP*, *bcf*, *stc*, *STY1358-67*, *STY1867-68*, SPI-4, *STY4842-43*, *gppA*, *mlc*, *typA*, *exbDB*, *STY1398*, and *STY2346* showed a slightly higher sensitivity relatively to the wild-type strain. These higher sensitivities were significant for mutants *bcf*, *STY1358-67*, SPI-4, *gppA*, and *mlc* (*P*<0.05) (Table 1).

### Interaction of deletion mutants with macrophages during competitive assay

**Figure 2:**
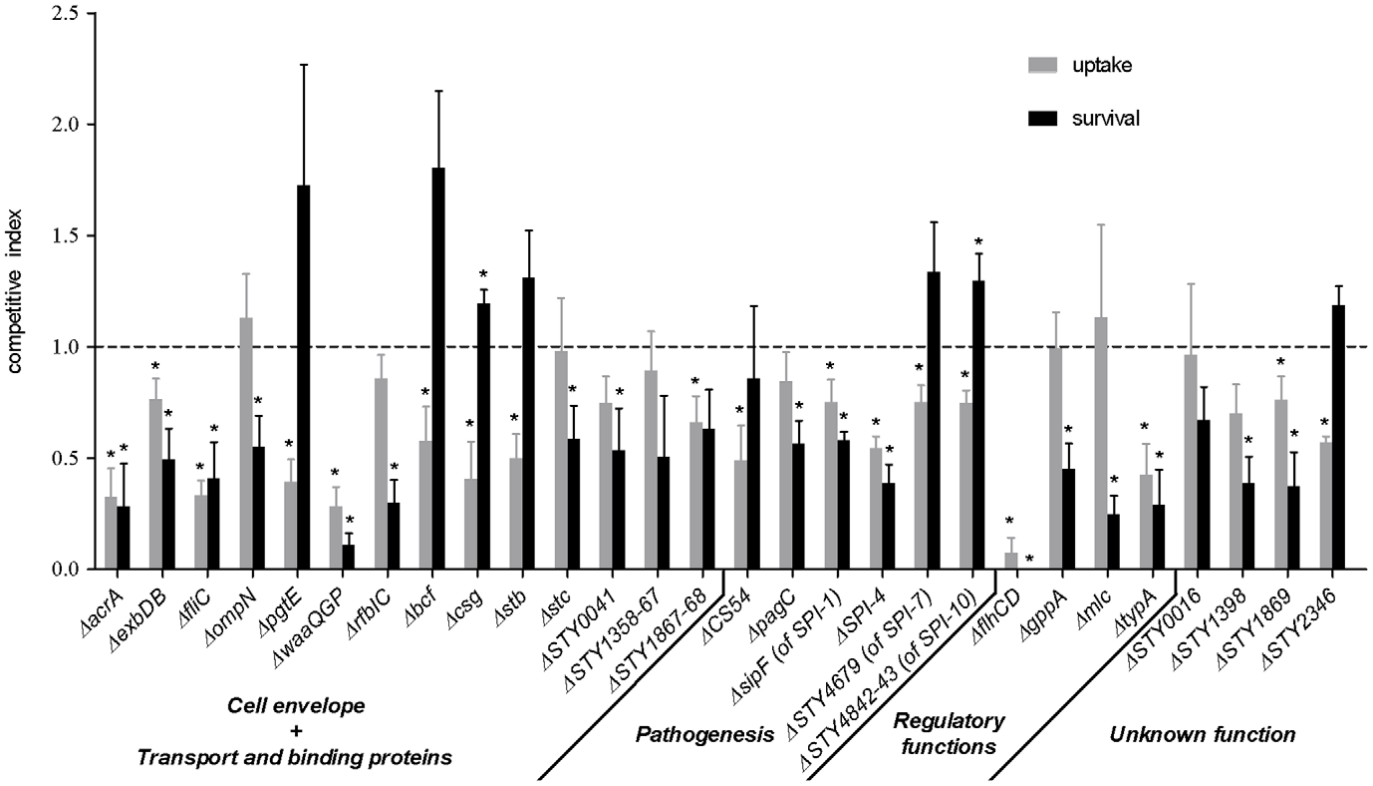
Uptake and intracellular survival of mutants within macrophages during the mixed infection assay. Competitive index (CI) assays for uptake (0 h) and intracellular survival (24 h post-infection) against the nalidixic acid-resistant wild-type *S*. Typhi (DEF566) were performed for all 28 isogenic mutants during infection of cultured THP-1 human macrophages. Functional classes are indicated below the gene names. Data presented are the mean ± standard error of the mean of at least three independent experiments performed in duplicate. Asterisks (*) represent CI values for mutants which are significantly different from 1 (*P*<0.05).

During the initial screening through macrophages, transposon mutants from the library were competing against each other while entering into and replicating inside the cells. Hence, the deletion mutants were tested using a competitive assay, where a Nal^r^ isogenic *S*. Typhi strain (DEF566) was used as the wild-type counterpart. CI values of mutants upon uptake (0 h) and during survival (24 h post-infection) were obtained. Nine mutants had a significantly lower CI upon uptake by macrophages (*pgtE*, *bcf*, *csg*, *stb*, *STY1867-68*, CS54, *STY4679*, *STY4842-43*, and *STY2346*), and eight mutants were significantly less competitive during intramacrophage survival (*ompN*, *rfbIC*, *stc*, *STY0041*, *pagC*, *gppA*, *mlc*, and *STY1398*) (*P*<0.05) (Figure 2). Furthermore, nine mutants were significantly outcompeted by the wild-type for both uptake and survival within macrophages (*acrA*, *exbDB*, *fliC*, *waaQGP*, *sipF*, SPI-4, *flhCD*, *typA*, and *STY1869*) (*P*<0.05) (Figure 2).

Mutants highly defective during interaction with macrophages, such as *fliC*, *waaQGP*, and *flhCD*, and those representing genes of unknown function, such as *STY1398*, *STY1869*, and *STY2346*, were complemented by a wild-type copy of the gene(s). These strains were used for competitive infection of macrophages with their corresponding mutants. The competition between the complemented strains and their mutants reproduced the phenotypes observed against the wild-type strain (Table 2). Complemented strains outcompeted their mutant counterparts at the uptake and survival stages, except for *waaQGP* at uptake (CI = 1.04) and *STY1398* during intracellular survival (CI = 1.19).

**Table 2:** Effect of plasmid-borne gene complementation on *S*. Typhi deletion mutants during competitive assays within macrophages.

	CI^a^ value for uptake		CI value for survival	
ORF(s) (gene name)	vs wild-type^b^	vs complemented mutant^c^	vs wild-type	vs complemented mutant
*STY2167* (*fliC*)	0.33 (±0.07) *^d^	0.42 (±0.09) *	0.41 (±0.16) *	0.10 (±0.07) *
*STY4071-73* (*waaQGP*)	0.28 (±0.09) *	1.04 (±0.14)	0.11 (±0.06) *	0.19 (±0.10) *
*STY2133-34* (*flhCD*)	0.07 (±0.07) *	0.16 (±0.05) *	0.00 (±0.00) *	0.07 (±0.07) *
*STY1398*	0.70 (±0.13)	0.97 (±0.19)	0.39 (±0.12)*	1.19 (±0.13)
*STY1869*	0.76 (±0.11) *	0.73 (±0.03) *	0.37 (±0.15) *	0.53 (±0.10) *
*STY2346*	0.57 (±0.03) *	0.67 (±0.12) *	1.19 (±0.09)	0.76 (±0.12)

a CI, competitive index.

b The wild-type counterpart is represented by the nalidixic acid-resistant wild-type *S*. Typhi (DEF566).

c Complemented mutants all carry the low-copy-number cloning vector pWSK29 [36] harbouring the respective deleted gene or gene cluster.

d Data presented are the mean ± standard error of the mean of at least three independent experiments performed in duplicate, where the deletion mutants were mixed either with the wild-type strain or the respective complemented mutant during infection of human macrophages. Asterisks (*) represent CI values for mutants which are significantly different from 1 (*P*<0.05).

### Interaction of deletion mutants with macrophages tested individually

**Figure 3:**
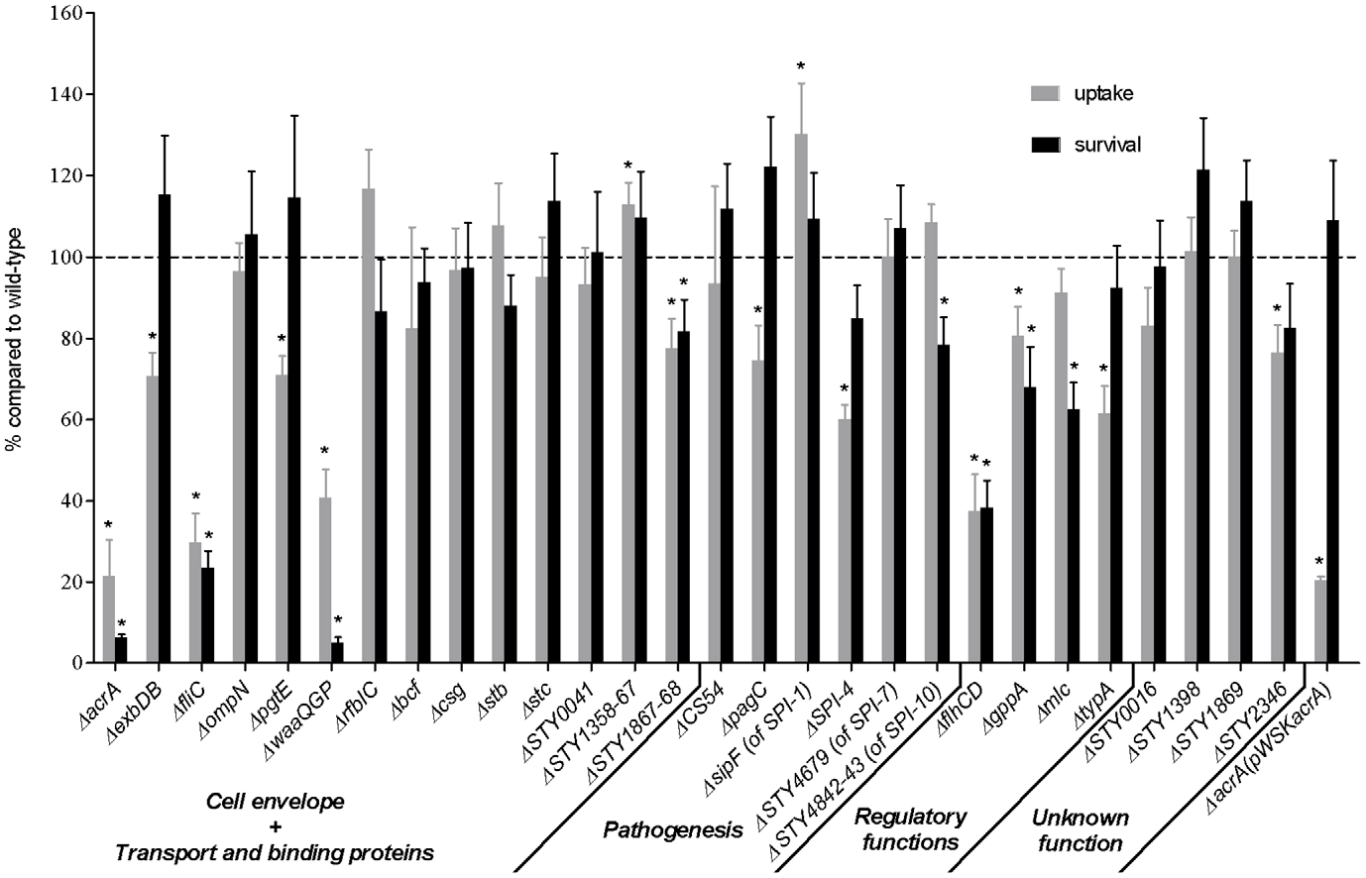
Uptake and intracellular survival rates of mutants tested individually in macrophages. THP-1 human macrophages were infected with *S*. Typhi wild-type strain ISP1820, all 28 isogenic mutants, and the complemented Δ*acrA*(pWSK*acrA*) strain. The number of intracellular bacteria was determined upon uptake (0 h) and during survival (24 h post-infection) within macrophages. Functional classes are indicated below the gene names. The values for percent recovery were normalized to the wild-type control value, defined as 100% at each time point. Data presented are the mean ± standard error of the mean of at least three independent experiments performed in duplicate. Asterisks (*) represent percentages for mutants which are significantly different from the isogenic wild-type (*P*<0.05).

The deletion mutants were individually assessed for their ability to infect macrophages. Hence, the rates of uptake and intracellular survival within human macrophages were determined for all the *S*. Typhi mutants, and compared with those of the isogenic wild-type strain, tested in parallel. Six mutants showed significantly lower uptake by macrophages (*exbDB*, *pgtE*, *pagC*, SPI-4, *typA*, and *STY2346*) and two mutants showed significantly lower intracellular survival (*STY4842-43* and *mlc*) (*P*<0.05) (Figure 3). Moreover, six mutants had a global interaction defect towards macrophages, with significant attenuation phenotypes observed both upon uptake and during survival (*acrA*, *fliC*, *waaQGP*, *STY1867-68*, *flhCD*, and *gppA*) (*P*<0.05) (Figure 3). The strongly attenuated mutant *acrA* was complemented with wild-type *acrA* (pWSK*acrA*). The significant survival defect within macrophages (*P*<0.05) was reversed in this complemented strain, as intracellular growth from 0 to 24 h post-infection was equivalent to that of the wild-type strain (Figure 3), hence confirming that the growth defect observed for the mutant was due to inactivation of *acrA*.

Overall, infection results of deletion mutants revealed that several mutants had a significant defect only during the competition experiment, including eight mutants during uptake (*bcf*, *csg*, *stb*, CS54, *sipF*, *STY4679*, *STY4842-43*, and *STY1869*) and 11 mutants defective in survival (*exbDB*, *ompN*, *rfbIC*, *stc*, *STY0041*, *pagC*, *sipF*, SPI-4, *STY1398*, *typA*, and *STY1869*) (*P*<0.05) (Figures 2 and 3). Interestingly, 12 mutants were significantly attenuated when subjected to both individual and competitive experiments, with 10 mutants in uptake (*acrA*, *exbDB*, *fliC*, *pgtE*, *waaQGP*, *STY1867-68*, SPI-4, *flhCD*, *typA*, and *STY2346*), and six mutants in survival (*acrA*, *fliC*, *waaQGP*, *flhCD*, *gppA* and *mlc*) (*P*<0.05) (Figures 2 and 3).

## Discussion

One of the key virulence features of *Salmonella* is its ability to survive inside macrophages, enabling the pathogen to systemically infect its animal [4] or human host [54]. To better understand how *S*. Typhi adapts to the macrophage environment, a comprehensive method was applied to screen for candidate genes used by *S*. Typhi during infection of human macrophages. Serial competitive passages of a transposon mutant library through macrophages led to the identification of 130 genes, belonging to 13 functional classes (Figure 1). Some of these genes were previously identified following screening of *S*. Typhi mutant pool within humanized mice (*STY0016*, *STY0039*, *STY2607*, *ydhO* and *STY4458*) [55] and antibodies against some of their products (CdtB, AcrA, PagC, PflB, STY1364, members of the *bcf*, *csg*, *stb* fimbrial operons and of the CS54 island) were detected in blood from typhoid patients [56]–[58], suggesting the importance of these proteins during *S*. Typhi systemic infection of its human host.

Surprisingly, comparison of genes selected inside human macrophages with transcriptomic data obtained at 24 h post-infection [33] revealed that among the 130 genes identified, only 8% (10 genes) were also upregulated inside these cells. A lack of correlation between expression and a fitness role for genes inside macrophages was also observed with the human-adapted pathogen *Mycobacterium tuberculosis* [59]. This suggests that bacterial genes involved in interaction with macrophages are not necessarily overexpressed at 24 h post-infection, but may be already expressed upon uptake or earlier during intracellular survival.

We constructed 28 isogenic markerless deletion mutants representing genes or gene clusters that belong to the five major functional classes (Figure 1). Competitive and individual infection assays were then conducted with these mutants to better determine involvement of genes during interaction with human macrophages. Using these assays, 35 defective phenotypes of either uptake or intracellular survival within macrophages were observed for the mutants (Table 1), all previously unassociated to the *Salmonella* genes studied here. 26 mutants were less competitive than the wild-type strain during either uptake into cells, intracellular survival or both of these processes (Figure 2), corroborating the selection of genes during the competitive screening strategy. Among these 26 mutants outcompeted by the wild-type strain during infection, six were resubmitted to competition experiments against their respective mutant bearing the plasmid-borne wild-type gene or gene cluster (Table 2). All mutant defects initially identified against the wild-type counterpart were maintained (Table 2), thus directly linking the genes complemented here to the attenuation phenotypes in intracellular survival observed during competition, with the exception of *STY1398*. The reasons explaining the failure to complement the *STY1398* mutant may result from plasmid loss or imbalance in gene copy-number. Moreover, when mutants were tested individually, a significant defect was observed in half of them (14 of 28 mutants) either upon uptake and/or during intracellular survival (Figure 3). Hence, the competitive assay was more sensitive than the individual infection assay in determining mutant impairment during bacterial infection of macrophages. Noticeable is that all the fimbrial mutants showed involvement only when under competition against the wild-type, either upon uptake (*bcf*, *csg*, *stb*) or during intracellular survival (*stc*) (Figure 2). In contrast, our results show a few examples of attenuation only when mutants were subjected to individual infection assay. This is possible if a mutant impaired when tested alone is rescued *in trans* by a product of the wild-type strain during the competitive assay, a phenomenon previously described in competition experiments [23], [26]. This was observed for mutants *pagC* and *gppA* upon entry in macrophages and mutants *STY1867-68* and *STY4842-43* during intracellular survival (Figures 2 and 3). For example, the PagC protein is important for induction of membrane vesicles released by *S*. Typhimurium in the extracellular environment [60]. This suggests that presence of vesicles produced by the wild-type strain in the surroundings of the *pagC* mutant could overcome its inability to secrete vesicle-associated virulence factors participating in macrophage infection. Only mutants *STY0016* and *STY1358-67* showed no significant attenuation when tested in competition or individually. However, phenotypes of impairment upon uptake or during intracellular survival may possibly be revealed only following multiple serial passages through macrophage infection assays, similar to the initial screening.

Among the deletion mutants, several defects of uptake and/or survival during infection of macrophages were observed for the first time in *Salmonella* (Table 1). For instance, mutation of *exbDB* in *S*. Typhi impaired both uptake and survival in macrophages (Figures 2 and 3). ExbD and ExbB interact with TonB, forming a complex transducing energy to outer membrane transporters. The *exbDB* mutant was probably affected in *tonB*-dependant substrate acquisition inside human macrophages, however the reason explaining the entrance defect remains unclear. The efflux pump component AcrA [61], [62] promoted *S*. Typhi uptake, which is similar to that observed in *S*. Typhimurium [63], but was also involved in survival up to 24 h post-infection inside human macrophages (Figures 2 and 3). Previous transcriptomic results have shown that inactivation of *acrA* in *S*. Typhimurium decreased expression of *phoP* [64], part of the two-component *phoPQ* system playing a key role in intracellular survival of *Salmonella* inside macrophages, thus explaining attenuation of the *S*. Typhi *acrA* mutant within macrophages observed here. Furthermore, an *S*. Typhimurium *acrA* mutant was affected in growth under anaerobic conditions after 24 h [64]. Hence, since the *Salmonella*-containing vacuole within infected cells is considered hypoxic [65], it can be postulated that inactivation of *acrA* in the *S*. Typhi mutant is in fact impairing the ability of the bacteria to replicate under such anaerobic conditions. However, it is less clear why the mutant is defective in uptake inside macrophages, although it has been proposed that lack of *acrA* creates membrane instability, which may confer poor entrance inside eukaryotic cells [64]. Although the *acrA* mutant was less resistant to 0.1% DOC in PBS over 2 h (10% mortality), bacteria were exposed to this compound for a much shorter time period following macrophage cell lysis when conducting infection assays. Moreover, the mortality rate of the mutant during intracellular survival in macrophages was much higher (approximately 80% mortality), hence its defect within macrophages was not attributable to a strong survival defect in DOC. Another explanation may be that membrane instability renders the *acrA* mutant more sensitive to antimicrobial peptides of the macrophage. As observed, the attenuated survival phenotype of the mutant was complemented by a plasmid-borne wild-type copy of the gene (Figure 3). Mutants with transposons in ORFs *STY1867*, *STY1868*, and *STY1869* were selected during screening (Table S3). *STY1867* and *STY1868* are roughly 150 bp apart in the *S*. Typhi genome and perhaps part of an operon, whereas *STY1869* is divergently transcribed [66]. *STY1867* encodes for a putative lipoprotein [66] that could be mediating attachment of *S*. Typhi to macrophage surfaces, since uptake of mutant *STY1867-68* was affected (Figures 2 and 3). *STY1868*, annotated as a putative cytochrome [66], is predicted to be an inner membrane protein [67]. Both *STY1868* and *STY1869* are of unknown function [66]. However, the *S*. Typhimurium homologue of *STY1868* (*STM1253*) is induced by the PmrA/PmrB two-component system involved in resistance to antimicrobial peptides [68], [69], and along with the *STY1869* homologue (*STM1252*), both ORFs are induced by the PreA/PreB two-component system, which itself regulates PmrA/PmrB and promotes invasion of human epithelial cells by *S*. Typhimurium [70], [71]. Sequence comparison of these homologous ORFs revealed a high degree of conservation between promoter regions of both serovars. Hence, it can be speculated that ORFs *STY1868* and *STY1869* from *S*. Typhi are regulated similarly to those of *S*. Typhimurium. However, an *STM1253* mutant showed no virulence defect in the mouse [68]. In *S*. Typhi, these ORFs may be mediating uptake and survival inside macrophages as part of the PreA/PreB regulon. The intramacrophage survival impairment of mutant *STY1867-68* could only be observed when tested individually and not when submitted to the competitive assay (Figures 2 and 3). Thus, this suggests that the products of *STY1867-68* in the wild-type are able to largely alleviate defects in these genes, *in trans*.

Many of the underrepresented genes were located on SPIs. SPI-1 encodes a T3SS involved in invasion of non-phagocytic epithelial cells [72], [73]. However, SPI-1 transposon mutants *hilC*, *prgI*, *sipC*, *sipF*, *spaS*, and *ygbA* were underrepresented after passages in macrophages (Table S3). Deletion of *sipF* (*iacP*) in *S*. Typhi impaired uptake and survival within macrophages (Figure 2). The *iacP* gene has been previously associated with invasiveness of chicks by *Salmonella enterica* serovar Enteritidis (*S*. Enteritidis) [74] and with invasion of epithelial cells by *S*. Typhimurium [75], phenotypes in conformity with the recognized operating functions of SPI-1. *iacP* was also associated with virulence of *S*. Typhimurium during infection of the mouse [25], [75]. Additionally, several genes from SPI-1 in *S*. Typhi were detected as virulence determinants when a transposon mutant pool was screened within a humanized mouse model [55]. Together, our results suggest that SPI-1 genes could represent *S*. Typhi virulence factors participating in optimal infection of human macrophages. SPI-2 encodes a T3SS involved in intracellular survival and systemic disease of *S*. Typhimurium. Genes *ssaN*, *ssaP*, *ssaQ*, and *orf408* from SPI-2 were identified during our screening (Table S3). However, a *ssaP* mutant replicates as much as the wild-type in human macrophages (data not shown), which correlates with previous data where complete deletion of SPI-2 T3SS was not involved in intracellular survival of *S*. Typhi in human macrophages [8]. We noticed that input values from these SPI-2 genes were higher than the average input intensity and that their output values were similar to the average output intensity while remaining higher than those of the other underrepresented genes (data not shown). SPI-4 encodes a T1SS for a non-fimbrial adhesin, that promotes adhesion to epithelial cells [76]. Since many SPI-4 genes were detected during initial screening (Table S3), the entire SPI-4 region was deleted and when tested, the mutant showed impaired uptake (Figures 2 and 3) and survival within macrophages (Figure 2). SPI-4 was initially thought to contribute to *S*. Typhimurium survival within mouse macrophages as indicated by screening of a transposon mutant bank [4], [77], but further research found no such involvement [76], [78], [79]. Interestingly, the transposon mutant of SPI-4 gene *STY4458* was underrepresented following competitive passage of an *S*. Typhi mutant pool in a humanized mouse infection model, and in our screening (Table S3) [55]. Hence, our results reiterate a potential intracellular role played by SPI-4, shown here specifically with serovar Typhi inside macrophages, but also identifies a role concerning uptake inside these cells (Figures 2 and 3). SPI-7 and -10 are both unique to *S*. Typhi when compared to *S*. Typhimurium [15]. Mutant *STY4679*, from SPI-7, and *STY4842-43*, from SPI-10, showed defects in uptake when under competition with the wild-type (Figure 2). Furthermore, the SPI-10 mutant was also attenuated during intramacrophage survival when tested individually (Figure 3).

Of genes with regulatory functions identified during screening (Figure 1 and Table S3), we tested mutants representing *flhCD*, *mlc*, *typA* and also *gppA* (for which the transposon mutant was strongly underrepresented (
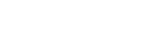
), although not significantly (*P*-value of 0.008). All these mutants showed defective phenotypes during infection assays with human macrophages that were described here for the first time (Table 1). Among other selected regulators (Table S3), the LysR-family regulator *ybdO* was detected and previously identified during screening of *S*. Typhimurium mutants underrepresented in murine macrophages [24] and in a mouse model of infection [25]. The deletion of *typA* affects uptake as well as intracellular survival (Figures 2 and 3). TypA (or BipA) is a translational GTPase [80] that regulates virulence mechanisms in *E. coli* [81], [82]. It is involved in flagella-associated motility and growth below 30°C [41], [42] and we have observed similar phenotypes in *S*. Typhi (Table 1; data not show). It also mediates resistance to certain antimicrobial peptides in *S*. Typhimurium and *E. coli* [81], [83] and was detected as a gene potentially involved in virulence of *S*. Typhimurium within a mouse model of infection [26]. Our work attributes a novel role to this regulatory element, linked for the first time to macrophage infection. Nonetheless, its precise functions concerning *Salmonella* virulence remain unclear, as more work is required to gain insight on its controlled regulon. The GppA enzyme hydrolyzes guanosine pentaphosphate (pppGpp) to guanosine tetraphosphate (ppGpp) rapidly *in vivo* [84], [85]. These are the two signal molecules mediating the stringent response [86]. Defective (p)ppGpp production causes impaired invasion and intracellular growth of *S*. Typhimurium in mouse macrophages [87], [88], and diminishes invasion and intracellular growth of *Salmonella enterica* serovar Gallinarum in murine and avian macrophages [89]. Thus, we propose that the stringent response, in which *gppA* takes part, is also required for full virulence of *S*. Typhi, and our novel results imply that GppA is involved in interaction of *Salmonella* with macrophages, since the mutant was attenuated both for uptake and intramacrophage survival (Figures 2 and 3).

In addition to genes with novel roles within macrophages described above, we have also confirmed defective phenotypes for mutations of genes that have been previously identified in other *Salmonella* serovars, such as those associated with LPS and flagella biosynthesis. For example, *waaG* is involved in linking the outer core to the inner core of LPS [90], [91], and when the *waaQGP* cluster was inactivated, uptake by human macrophages was lower compared to the wild-type strain (Figure 3), whereas a *waaG* (*rfaG*) mutant in *S*. Typhimurium shows higher uptake in murine macrophages [92], [93]. However, intracellular survival in macrophages of these mutants is lower for both serovars (Figure 3) [92], [93]. It is noteworthy that the *rfbIC* mutant, encoding genes involved in O-Ag biosynthesis [94], was outcompeted by the wild-type strain 24 h post-infection (Figure 2). This result corroborates the previously shown potential involvement of *rfbI* from *S*. Enteritidis during interaction with chicken macrophages, observed by screening of a mutant library [95]. Involvement of surface LPS during *S*. Typhi infection of human macrophages was further demonstrated by identification of outer core biosynthesis genes *waaI* and *waaK*, and the O-Ag ligase gene *waaL* (Table S3). A *waaL* mutant in *S*. Typhimurium is affected in intracellular growth inside murine macrophages [92], [93]. Thus, with the exception that the outer core seems to differentially influence entry into macrophages of serovars Typhi and Typhimurium, the survival phenotypes observed here concerning our LPS-associated mutants in *S*. Typhi are similar to those previously observed with other *Salmonella* serovars.

Strong attenuation was observed for flagellar mutants *flhCD* and *fliC* during macrophage infection (Figures 2 and 3). In *S*. Typhimurium, a higher replication of *flhD* and *fljB fliC* mutants within mouse macrophages was observed [96]. In *S*. Enteritidis, a *fliC* mutant was deficient in entry into porcine blood monocytes, similar to *S*. Typhi results in human macrophages (Figure 3) [97]. *flgI* and *fliD* were also identified during our screening (Table S3). The *fliD* locus in *S*. Typhimurium [4], [77] and *flgI*, *fliD*, *flhC* and *flhD* in *S*. Enteritidis [95] were identified upon passages of mutant libraries within macrophages. Nonetheless, our study has highlighted for the first time involvement of flagellar genes during intracellular survival of serovar Typhi within human macrophages.

In conclusion, screening of a *S*. Typhi transposon mutant library through cultured human macrophages for 24 h selected mutants with less fitness in these cells and revealed 130 genes potentially involved in interaction with macrophages. Among the defined deletion mutants representing selected genes, a great majority were significantly defective for uptake and/or intracellular survival inside macrophages during competitive and individual infection assays. Many of these were genes not previously known to contribute to entry and to intracellular survival of serovar Typhi within human macrophages, including cell envelope components, SPI-encoded features, regulatory elements and many ORFs of unknown function (Table 1). Furthermore, as attenuation of *S*. Typhi replication inside macrophages has been associated with avirulence within human beings [54], *S*. Typhi genes involved in infection of human macrophages represent potential targets for improvement or development of typhoid fever therapies.

## Supporting Information

Table S1 **Bacterial strains and plasmids used in this study.**
(PDF)

Table S2 **Primers used in this study.**
(PDF)

Table S3 **List of** ***S*** **. Typhi genes identified following negative selection of mutant pool in human macrophages.**
(PDF)
