# Supplementary material for: Selection of Salmonella enterica Serovar Typhi Genes Involved during Interaction with Human Macrophages by Screening of a Transposon Mutant Library
Source: PLoS One. 2012 May 4;7(5):e36643. doi: 10.1371/journal.pone.0036643 (PMC3344905; doi:10.1371/journal.pone.0036643)
Supplement: Table S1 — Bacterial strains and plasmids used in this study. (PDF) [file pone.0036643.s001.pdf]

Table S1. Bacterial strains and plasmids used in this study.

Strains	Characteristics	Source or Reference[a]
S. Typhi		
ISP1820	Wild-type *S*. Typhi	[1]
DEF566	ISP1820 Nal[r]	[2]
DEF435	ISP1820 Δ*fliC*	This study
DEF496	ISP1820 Δ*pgtE*	This study
DEF510	ISP1820 Δ*csg*	This study
DEF511	ISP1820 Δ*pagC*	This study
DEF512	ISP1820 Δ*typA*	This study
DEF534	ISP1820 Δ*mlc*	This study
DEF535	ISP1820 ΔSTY1358-67	This study
DEF541	ISP1820 Δ*exbDB*	This study
DEF544	ISP1820 Δ*waaQGP*	This study
DEF546	ISP1820 ΔCS54	This study
DEF547	ISP1820 ΔSTY1867-68	This study
DEF548	ISP1820 ΔSTY1869	This study
DEF555	ISP1820 Δ*acrA*	This study
DEF556	ISP1820 ΔSTY1398	This study
DEF557	ISP1820 Δ*ompN*	This study
DEF560	ISP1820 Δ*rfbIC*	This study

DEF561	ISP1820 Δ*sipF*	This study
DEF562	ISP1820 ΔSTY0041	This study
DEF563	ISP1820 Δ*bcf*	This study
DEF564	ISP1820 Δ*stb*	This study
DEF571	ISP1820 Δ*flhCD*	This study
DEF601	ISP1820 Δ*gppA*	This study
DEF602	ISP1820 Δ*stc*	This study
DEF625	ISP1820 ΔSTY0016	This study
DEF627	ISP1820 ΔSTY2346	This study
DEF628	ISP1820 ΔSPI-4	This study
DEF632	DEF555 (pWSK*acrA*)	This study
DEF646	DEF435 (pWSK*fliC*)	This study
DEF656	ISP1820 ΔSTY4679	This study
DEF662	ISP1820 ΔSTY4842-43	This study
DEF766	DEF571 (pWSK*flhCD*)	This study
DEF767	DEF544 (pWSK*waaQGP*)	This study
DEF768	DEF556 (pWSKSTY1398)	This study
DEF769	DEF548 (pWSKSTY1869)	This study
DEF770	DEF627 (pWSKSTY2346)	This study
E. coli		
MGN-617	SM10 λpir *asd thi thr leu tonA lacY supE recA* RP4 2-Tc::Mu[λpir] Δ*asdA4*	[3]

Plasmids

pLOFKm	mini-Tn*10*-Km-based delivery plasmid with IPTG-inducible IS10_R transposase driven by p*tac* promoter, *lacI*^{q} *mobRP4 ori*R6K; Km^r Ap^r	[4]
pLOFKm-T7 (pSIF117)	pLOFKm with T7 RNA-polymerase promoter *Not*I-inserted in 3' region of mini-Tn*10*	This study
pMEG-375	*sacRB mobRP4 ori*R6K; Cm^r Ap^r	R. Curtiss III, ASU
pWSK29	Low-copy-number cloning vector; Ap^r	[5]
pWSK*acrA*	pWSK29 carrying *acrA*	This study
pWSK*fliC*	pWSK29 carrying *fliC*	This study
pSIF113	pMEG-375 with flanking region of *fliC* used for *fliC* deletion	This study
pSIF133	pMEG-375 with flanking region of *pgtE* used for *pgtE* deletion	This study
pSIF143	pMEG-375 with flanking region of *csg* operon used for *csg* operon deletion	This study
pSIF144	pMEG-375 with flanking region of *pagC* used for *pagC* deletion	This study
pSIF145	pMEG-375 with flanking region of *typA* used for *typA* deletion	This study
pSIF156	pMEG-375 with flanking region of *mlc* used for *mlc* deletion	This study

pSIF157	pMEG-375 with flanking region of *exbDB* used for *exbDB* deletion	This study
pSIF159	pMEG-375 with flanking region of STY1358-67 used for STY1358-67 deletion	This study
pSIF160	pMEG-375 with flanking region of *waaQGP* used for *waaQGP* deletion	This study
pSIF162	pMEG-375 with flanking region of STY1869 used for STY1869 deletion	This study
pSIF163	pMEG-375 with flanking region of STY1867-68 used for STY1867-68 deletion	This study
pSIF164	pMEG-375 with flanking region of CS54 used for CS54 deletion	This study
pSIF165	pMEG-375 with flanking region of *acrA* used for *acrA* deletion	This study
pSIF166	pMEG-375 with flanking region of STY1398 used for STY1398 deletion	This study
pSIF167	pMEG-375 with flanking region of *ompN* used for *ompN* deletion	This study
pSIF171	pMEG-375 with flanking region of STY0041 used for STY0041 deletion	This study
pSIF173	pMEG-375 with flanking region of *sipF* used for *sipF* deletion	This study
pSIF174	pMEG-375 with flanking region of *rfbIC* used	This study

	for *rfbIC* deletion	
pSIF175	pMEG-375 with flanking region of *bcf* operon used for *bcf* operon deletion	This study
pSIF176	pMEG-375 with flanking region of *stb* operon used for *stb* operon deletion	This study
pSIF184	pMEG-375 with flanking region of *flhCD* used for *flhCD* deletion	This study
pSIF198	pMEG-375 with flanking region of *stc* operon used for *stc* operon deletion	This study
pSIF204	pMEG-375 with flanking region of *gppA* used for *gppA* deletion	This study
pSIF212	pMEG-375 with flanking region of SPI-4 used for SPI-4 deletion	This study
pSIF213	pMEG-375 with flanking region of STY4842-43 used for STY4842-43 deletion	This study
pSIF214	pMEG-375 with flanking region of STY2346 used for STY2346 deletion	This study
pSIF215	pMEG-375 with flanking region of STY0016 used for STY0016 deletion	This study
pSIF217	pMEG-375 with flanking region of STY4679 used for STY4679 deletion	This study
pSIF285	pWSK29 carrying *flhCD*	This study
pSIF286	pWSK29 carrying *waaQGP*	This study

pSIF287	pWSK29 carrying STY1398	This study
pSIF288	pWSK29 carrying STY1869	This study
pSIF289	pWSK29 carrying STY2346	This study

[a]References:

1. Hone DM, Harris AM, Chatfield S, Dougan G, Levine MM (1991) Construction of genetically defined double *aro* mutants of *Salmonella typhi*. Vaccine 9: 810-816.

2. Forest CG, Ferraro E, Sabbagh SC, Daigle F (2010) Intracellular survival of *Salmonella enterica* serovar Typhi in human macrophages is independent of *Salmonella* pathogenicity island (SPI)-2. Microbiology 156: 3689-3698.

3. Kaniga K, Compton M, Curtiss III R, Sundaram P (1998) Molecular and functional characterization of *Salmonella enterica* serovar typhimurium *poxA* gene: effect on attenuation of virulence and protection. Infect Immun 66: 5599-5606.

4. Herrero M, de Lorenzo V, Timmis K (1990) Transposon vectors containing non-antibiotic resistance selection markers for cloning and stable chromosomal insertion of foreign genes in gram-negative bacteria. J Bacteriol 172: 6557-6567.

5. Wang RF, Kushner SR (1991) Construction of versatile low-copy-number vectors for cloning, sequencing and gene expression in *Escherichia coli*. Gene 100: 195-199.
