# Supplementary material for: Selection of Salmonella enterica Serovar Typhi Genes Involved during Interaction with Human Macrophages by Screening of a Transposon Mutant Library
Source: PLoS One. 2012 May 4;7(5):e36643. doi: 10.1371/journal.pone.0036643 (PMC3344905; doi:10.1371/journal.pone.0036643)
Supplement: Table S2 — Primers used in this study. (PDF) [file pone.0036643.s002.pdf]

Table S2. Primers used in this study.

Primers	Sequence (5’–3’)[a]
Front NotI-Kan	AAGGAAAAAAGCGGCCGCGCCGTCCCGTCAAGTCAGCG
Rear NotI-Kan	AAGGAAAAAAGCGGCCGC**CCCTATAGTGAGTCGTATTA**CTCTGATGTTACATTGCACAAG
pLOF F seq	CAAGACGTTTCCCGTTGAAT
STY:PCRNiche#2	CTTGTGCAATGTAACATCAGAG
CCT$_{24}$VN	CCTTTTTTTTTTTTTTTTTTTTTTTTVN
STY0016 F1	CGGGATCCGTGATATCGCCATTATCGCG
STY0016 R2	*TGAGGGCTT*CGCCTTCAAGCGCAATAATT
STY0016 F3	*TTGAAGGCG*AAGCCCTCAATTGCAGTCAG
STY0016 R4	AAGGAAAAAAGCGGCCGCATATCAGGCTCATCCGTGAG
STY0041 F1	CGGGATCCACCTCATGGAGTTGAGCATG
STY0041 R2	*GGTCGCCGT*AGTCGCCAT ATCCGAAGCTA
STY0041 F3	*ATGGCGACT*ACGGCGACCGTATGAATTAC
STY0041 R4	AAGGAAAAAAGCGGCCGCACAAACCAATGCACAGCGAC
STY1358-67 F1	CGGGATCCGTGTACACATTGCTTGCCGA
STY1358-67 R2	*TTCGATGAT*GAATACTGCCGGATGTAGAGAG
STY1358-67 F3	*CGGCAGTAT*TCTCATCGAAGCATGGACT
STY1358-67 R4	AAGGAAAAAAGCGGCCGCCCATGTTAACGCTGGCGATG
STY1398 F1	CGGGATCCGATGAATTGTACGCGGCAGA
STY1398 R2	*TGCCAGCGG*CTCAATCCAGCCATCCAGTT

STY1398 F3	*TGGATTGAG*CCGCTGGCATCATTTTCAGT
STY1398 R4	AAGGAAAAAAGCGGCCGCGCATGTTAGTGTACCACGAG
STY1398 compl R	CCGCTCGAGGCATGTTAGTGTACCACGAG
STY1649 F1	CGGGATCCAGGATGCCTTTGATTCAACG (*ompN*)
STY1649 R2	*CGTCGTCAT*TCCCGTAAAGGTCCAGTTTG (*ompN*)
STY1649 F3	*TTACGGGAA*TGACGACGACCCGTTCTAT (*ompN*)
STY1649 R4	AAGGAAAAAAGCGGCCGCTGTCGGGAGATAAGCAAACC (*ompN*)
STY1867-68 F1	GCTCTAGACCATCAGGTACGACATCAAC
STY1867-68 R2	*GAGAAAAGT*CCGTATATAAGCCAATCCCT
STY1867-68 F3	*TATATACGG*ACTTTTCTCGCCTTACCGCT
STY1867-68 R4	AAGGAAAAAAGCGGCCGCAATGGTGAAACAACGCCGCT
STY1869 F1	CGGGATCCCTCTCGACATTCTGCCTGTT
STY1869 R2	*AACCAGATC*ATAGCGAGATAGCCAGATGG
STY1869 F3	*TCTCGCTAT*GATCTGGTTAGTATGCAGGAC
STY1869 R4	AAGGAAAAAAGCGGCCGCATTCCAGCGCTATCGGTTTC
STY1869 compl R	CCGCTCGAGATTCCAGCGCTATCGGTTTC
STY2346 F1	CGGGATCCTTCCTCACGTTTACCCTGTG
STY2346 R2	*ATCAACAAC*CAGCACGGTAAAGCAGTTGA
STY2346 F3	*ACCGTGCTG*GTTGTTGATCCTAACGTGGC
STY2346 R4	AAGGAAAAAAGCGGCCGCACGCAGATCCTTCAGACGAT
STY2346 compl R	CCGCTCGAGACGCAGATCCTTCAGACGAT
STY2378-81 F1	GCTCTAGATGTTGACTGCCTTCACTACC (*stc*)
STY2378-81 R2	*GCTGAAATT*AAGCGACTGCGCTGATCTAT (*stc*)

STY2378-81 F3	*CAGTCGCTT*AATTTCAGCGGTGTTCGTAC (*stc*)
STY2378-81 R4	AAGGAAAAAAGCGGCCGCGCGATAACTTCCTGTCTATG (*stc*)
STY3641 F1	CGGGATCCGTAATCGTCGTCGTTCAGGT (*gppA*)
STY3641 R2	*AACGTCAGA*TCAATGGCGGCATACAACGA (*gppA*)
STY3641 F3	*CGCCATTGA*TCTGACGTTAACCTTGCCTG (*gppA*)
STY3641 R4	AAGGAAAAAAGCGGCCGCGGCAAAGTCACCTGGCTAAT (*gppA*)
STY4452-60 F1	GCTCTAGACTATAAAGCGTATTGGTAGC (SPI-4)
STY4452-60 R2	*TGATCAACG*TCAACAAAACTAGGCCACGG (SPI-4)
STY4452-60 F3	*TTTTGTTGA*CGTTGATCATGGTGACGCAT (SPI-4)
STY4452-60 R4	AAGGAAAAAAGCGGCCGCTTGCCGACTCGTTAAATGAC (SPI-4)
STY4679 F1	CGGGATCCACGAATGATTGCCCACACAG
STY4679 R2	*TGACCCAAC*TTGAGGGCTTGGATGGTAAG
STY4679 F3	*AGCCCTCAA*GTTGGGTCAATCGTTCCTAT
STY4679 R4	AAGGAAAAAAGCGGCCGCCCACAGACTTCGGTTTTGGT
STY4842-43 F1	CGGGATCCACTCGAGTGACTCTGATGAA
STY4842-43 R2	*GACCCACAA*CCTCATTACGATATGGTCA
STY4842-43 F3	*TAATGAGGT*TGTGGGTCGGAAGCTGGATA
STY4842-43 R4	AAGGAAAAAAGCGGCCGCAGCCGGAATATTCCGGTCAT
acrA F1	CGGGATCCACGCAGCAATGGGTTTAAGG
acrA R2	*CCACTTATC*TTGCTGGTCCTGTTTGTCGT
acrA F3	*GACCAGCAA*GATAAGTGGCTGGTGACTGA
acrA R4	AAGGAAAAAAGCGGCCGCAAGTTAAGACCTGGGCTGAG
bcf F1	CGGGATCCACTCACGACGTTGAGTAGCT

bcf R2	*ACGTCATTC*TGACGGTTGTAGTATCCGCT
bcf F3	*CAACCGTCA*GAATGACG TGGGAACCTTAG
bcf R4	AAGGAAAAAAGCGGCCGCTGCTACGCGGTTCAGTCATA
CS54 F1	GCTCTAGACCTGCTGATTTAGAATTTGATCC
CS54 R2	*GAGTCACGC*GCAGGCTGCGTGAAATTAGA
CS54 F3	*GCAGCCTGC*GCGTGACTCGCTTTTACTTG
CS54 R4	CCCGGGGAGAACCCGTATGCGAACAA
csg F	CGGGATCCTGGGGCTAATCTTTGGCTAT
csg R over	*AACTCAATC*TATGAAGTACAGGCAGGCGT
csg F over	*TACTTCATA*GATTGAGTTGTCTCGTCTTA
csg R	AAGGAAAAAAGCGGCCGCGCCTATGGCAGGGATATTTT
exbD/B F1	CGGGATCCTGATGCAGACGGATCTTTCC
exbD/B R2	*ACTCTACGG*TTAAGCGGCGCTTTTGCGT
exbD/B F3	*GCCGCTTAA*CCGTAGAGTATGAAACCCTG
exbD/B R4	AAGGAAAAAAGCGGCCGCGGGAACATTTAGGCTAACGC
flhC/D F1	CGGGATCCCTATGACAGGATGCGCAGTC
flhC/D R2	*CAGCGTTTG*TTGCGTGTAGTTTATGCCAG
flhC/D F3	*TACACGCAA*CAAACGCTGTGCAAGGAGTA
flhC/D R4	AAGGAAAAAAGCGGCCGCTGGGAAGCTGCGTTATACGT
fliC F	CGGGATCCAAGCCATGCCTTCTTCCTTT
fliC R-over	*AGAGGACGT*ACAGCGACAGGCTGTTTGT
fliC F-over	*TGTCGCTGT*ACGTCCTCTCTTTACTGCGTTA
fliC R	GCTCTAGAGGGAAAGAGGAACAGGTATGA

LPS compl F	CGGGATCCCCCAGACAATGGGAATGACC
LPS compl R	CCGCTCGAGCCCCAATGATAAGAGTTACCT
mlc F1	CGGGATCCGCCTGGGCATATCGATCAAA
mlc R2	*CAGAATGCG*TTTGCTGCTAAGATCGCGCA
mlc F3	*AGCAGCAAA*CGCATTCTGGCTATCATGGT
mlc R4	AAGGAAAAAAGCGGCCGCCTCCACCGTTATGCTTCGAA
pagC F	CGGGATCCCATTGTAGAACCGGTTACCT
pagC R over	*CACCCGCGC*AATCCTGAACTTTACTTTGTG
pagC F over	*TTCAGGATT*GCGCGGGTGTACAGATGAAT
pagC R	AAGGAAAAAAGCGGCCGCATCACCTACCTGAGCAACCT
pgtE F	CGGGATCCATTATGATTATAGATTGCTTATT
pgtE R over	*ATAATATCC*GGTGTCGGTATCATAAACCA
pgtE F over	*ACCGACACC*GGATATTATATTACCAGTAATGC
pgtE R	AAGGAAAAAAGCGGCCGCCCACAAAGAGTATATGGAAAG
rfbI/C F1	CGGGATCCCCAGGATGACCGCTTTCATA
rfbI/C R2	*TTGGCGATG*TGTCCATCATGCTGTGATGG
rfbI/C F3	*TGATGGACA*CATCGCCAAAAACTTTAGGC
rfbI/C R4	AAGGAAAAAAGCGGCCGCATAAAACTGGCCGGGCCAT
sipF F1	CGGGATCCCCTTATCGGAGACGGTAGCT
sipF R2	*GAGCGAGAG*CATCAACGGCAATACAGGAG
sipF F3	*CCGTTGATG*CTCTCGCTCGACAACAATTG
sipF R4	AAGGAAAAAAGCGGCCGCCAGCAGTTCCTTCGGATCAT
stb F1	CGGGATCCTGCTGAATTCTGGCCTGTCT

stb R2	*GTTATTGCC*CGCCGAAAACAGCACTTGAT
stb F3	*TTTTCGGCG*GGCAATAACACGACGGGTTT
stb R4	AAGGAAAAAAGCGGCCGCCAGGAGGGTATAGCTCACAT
STY LPS F1	CGGGATCCATCAGCACGCTGAAGCAGAA (*waaQGP*)
STY LPS R2	*AACACGCTT*ACGATTACCGAAATCTTGCG (*waaQGP*)
STY LPS F3	*GGTAATCGT*AAGCGTGTTGCGACTATGGT (*waaQGP*)
STY LPS R4	AAGGAAAAAAGCGGCCGCATCTTTGTCACGCCAGCGAA (*waaQGP*)
typA F	CGGGATCCCAAAGAGACTCTGCAATACT
typA R over	*TCAGTTTCT*CAACGATGTTGATACGATAA
typA F over	*ACATCGTTG*AGAAACTGACCAACATGCGT
typA R	AAGGAAAAAAGCGGCCGCTCATTGGTCGGCATAATACG

[a]Restriction enzyme sites are underlined. Letters in italics denote overlapping sequences. Letters in bold represent the T7 RNA-polymerase promoter sequence. Corresponding gene names are given in parenthesis following sequence where necessary.
