# Supplementary material for: Selection of Salmonella enterica Serovar Typhi Genes Involved during Interaction with Human Macrophages by Screening of a Transposon Mutant Library
Source: PLoS One. 2012 May 4;7(5):e36643. doi: 10.1371/journal.pone.0036643 (PMC3344905; doi:10.1371/journal.pone.0036643)
Supplement: Table S3 — List of S . Typhi genes identified following negative selection of mutant pool in human macrophages. (PDF) [file pone.0036643.s003.pdf]

Table S3. List of *S*. Typhi genes identified following negative selection of mutant pool in human macrophages.

***Salmonella* Typhi**	***Salmonella* Typhi Name**	***Salmonella* Typhimurium**	***Salmonella* Typhimurium Name**	***Salmonella* Typhi Sanger Product**	**Functional class**	**log2 output/input**	***P*-value**
STY0015		STM0015		putative phage protein	Phage and insertion sequence elements	-2.54	0.00001
STY0016		STM0016		conserved hypothetical protein	Unknown	-3.13	0.00000
STY0021		STM0020		conserved hypothetical protein	Unknown	-3.57	0.00001
STY0031	*bcfF*	STM0026	*bcfF*	fimbrial subunit	Cell envelope	-2.12	0.00004
STY0039		STM0032		possible sulfatase	Small molecule metabolism	-4.00	0.00035
STY0040		STM0033		putative secreted 5'-nucleotidase	Unknown	-2.13	0.00005
STY0041		STM0034		putative exported protein	Cell envelope	-5.75	0.00001
STY0134	*leuO*	STM0115	*leuO*	probable activator protein in leuABCD operon	Amino acid biosynthesis/degradation	-2.69	0.00001
STY0207	*staA, yadN*			putative fimbrial protein	Cell envelope	-3.06	0.00002
STY0231	*htrA*	STM0209	*htrA*	protease DO precursor; heat shock protein HtrA	Protein biosynthesis/degradation	-3.69	0.00000
STY0329		STM0298		IS element transposase (pseudogene)	Phage and insertion sequence elements	-2.04	0.00015
STY0372	*stbB*	STM0339	*stbB*	fimbrial chaperone protein	Cell envelope	-3.69	0.00000
STY0419	*proC*	STM0386	*proC*	pyrroline-5-carboxylate reductase	Amino acid biosynthesis/degradation	-3.57	0.00009
STY0477		STM0437		conserved hypothetical protein	Unknown	-2.29	0.00001
STY0520	*acrA*	STM0476	*acrA*	acriflavin resistance protein A precursor	Cell envelope	-4.13	0.00014
STY0569	*ybbW*	STM0522	*ybbV*	putative allantoin permease (pseudogene)	Transport and binding proteins	-2.60	0.00011
STY0605		STM0557		putative membrane protein	Cell envelope	-4.55	0.00032
STY0651	*ybdO*	STM0606	*ybdO*	putative lysR-family transcriptional regulator	Regulatory functions	-2.91	0.00041
STY0804	*gpmA*	STM0772	*gpmA*	phosphoglycerate mutase 1	Energy metabolism	-4.71	0.00023
STY0878	*ybiS*	STM0837	*ybiS*	putative exported protein	Cell envelope	-4.52	0.00004
STY0917	*ulaA*	STM0884	*ulaA*	possible transport protein	Transport and binding proteins	-2.42	0.00008
STY0968	*pflA*	STM0970	*pflA*	pyruvate formate-lyase 1 activating enzyme	Energy metabolism	-4.12	0.00005
STY0973	*pflB*	STM0973	*pflB*	formate acetyltransferase 1	Energy metabolism	-6.02	0.00015
STY0974	*focA*	STM0974	*focA*	probable formate transporter (formate channel)	Transport and binding proteins	-6.17	0.00002

STY0977	*serC*	STM0977	*serC*	phosphoserine aminotransferase	Amino acid biosynthesis/degradation	-2.24	0.00005
STY1041				putative prophage membrane protein	Phage and insertion sequence elements	-2.33	0.00044
STY1121	*sigD, sopB*	STM1091	*sopB*	cell invasion protein	Pathogenesis	-2.08	0.00004
STY1129		STM1097		putative exported protein	Cell envelope	-2.57	0.00004
STY1157		STM1122	*ycdC*	putative transcriptional regulator	Regulatory functions	-3.16	0.00038
STY1177	*csgF*	STM1140	*csgF*	assembly/transport component in curli production	Cell envelope	-4.74	0.00041
STY1187	*mdoG*	STM1150	*mdoG*	periplasmic glucans biosynthesis protein MdoG precursor	Cellular processes	-3.16	0.00018
STY1220	*flgI*	STM1181	*flgI*	flagellar P-ring protein precursor	Cell envelope	-4.16	0.00032
STY1312	*cls*	STM1739	*cls*	cardiolipin synthetase	Cell envelope	-3.72	0.00039
STY1364				hypothetical periplasmic protein	Cell envelope	-3.47	0.00003
STY1397		STM1667		putative thiol peroxidase	Unknown	-2.33	0.00010
STY1398		STM1666		hypothetical protein	Unknown	-3.84	0.00001
STY1401		STM1663	*ynaI*	putative membrane protein	Cell envelope	-2.22	0.00044
STY1408		STM1657		putative chemo-receptor protein	Cellular processes	-2.79	0.00042
STY1427	*acpD*	STM1642	*acpD*	acyl carrier protein phosphodiesterase	Small molecule metabolism	-2.37	0.00044
STY1497	*osmC*	STM1563	*osmC*	osmotically inducible protein C	Cellular processes	-3.90	0.00019
STY1523	*hyaA2*	STM1539		uptake hydrogenase small subunit	Energy metabolism	-4.89	0.00007
STY1534		STM1527		putative membrane protein	Cell envelope	-2.02	0.00002
STY1554		STM1507	*yfdfJ*	putative membrane transport protein	Transport and binding proteins	-2.77	0.00021
STY1576		STM1488	*mlc*	putative regulatory protein	Regulatory functions	-2.25	0.00018
STY1585		STM1482	*ydgF*	putative conserved membrane protein	Cell envelope	-5.21	0.00002
STY1643				DNA-invertase	Phage and insertion sequence elements	-2.28	0.00008
STY1649		STM1473	*ompN*	outer membrane protein	Transport and binding proteins	-3.87	0.00005
STY1690		STM1432	*ydhO*	putative secreted protein	Cell envelope	-2.70	0.00003
STY1702	*ssaQ*	STM1418	*ssaQ*	putative type III secretion protein	Pathogenesis	-2.55	0.00048
STY1703	*ssaP*	STM1417	*ssaP*	putative type III secretion protein	Pathogenesis	-3.43	0.00028
STY1705	*ssaN*	STM1415	*ssaN*	putative type III secretion ATP synthase	Pathogenesis	-2.46	0.00023
STY1739		STM1382	*orf408*	Putative ribokinase (pseudogene)	Pathogenesis	-2.26	0.00022
STY1770	*btuC*	STM1340	*btuC*	vitamin B12 transport system permease	Transport and binding proteins	-2.25	0.00033
STY1852	*aroQ*	STM1269		putative chorismate mutase	Amino acid biosynthesis/degradation	-2.95	0.00001

STY1859		STM1261		conserved hypothetical protein	Unknown	-2.76	0.00024
STY1867		STM1254		putative lipoprotein	Cell envelope	-5.46	0.00018
STY1868		STM1253		putative cytochrome	Unknown	-4.14	0.00003
STY1869		STM1252		hypothetical protein	Unknown	-2.10	0.00042
STY1878	*pagC*	STM1246	*pagC*	outer membrane invasion protein	Pathogenesis	-2.23	0.00049
STY1886	*cdtB*			putative toxin-like protein	Pathogenesis	-3.86	0.00006
STY1910		STM1784	*ychF*	putative ATP/GTP-binding protein	Unknown	-3.28	0.00039
STY1976	*prc*	STM1845	*prc*	tail-specific protease precursor	Protein biosynthesis/degradation	-4.85	0.00016
STY2091	*kdgA*	STM1884	*eda*	multifunctional:2-keto-3-deoxygluconate 6-phosphate aldolase; 2-keto-4-hydroxyglutarate aldolase; oxaloacetate decarboxylase	Energy metabolism	-4.16	0.00003
STY2134	*flhD*	STM1925	*flhD*	flagellar transcriptional activator FlhD	Regulatory functions	-3.22	0.00043
STY2167	*fliC, flaG*	STM1959	*fliC*	flagellin	Cell envelope	-2.72	0.00000
STY2168	*fliD*	STM1960	*fliD*	flagellar hook associated protein 2	Cell envelope	-7.08	0.00009
STY2171	*amyA*	STM1963	*amyA*	cytoplasmic alpha-amylase	Macromolecule metabolism	-2.18	0.00042
STY2251	*pduK*	STM2046	*pduK*	putative propanediol utilization protein PduK	Small molecule metabolism	-2.10	0.00008
STY2276	*sbcB*	STM2067	*sbcB*	exodeoxyribonuclease I	DNA/RNA replication, degradation, modification	-2.04	0.00003
STY2289	*ugd, udg*	STM2080	*udg*	UDP-glucose 6-dehydrogenase	Small molecule metabolism	-2.54	0.00001
STY2303	*rfbI*	STM2093	*rfbI*	putative reductase RfbI	Cell envelope	-2.87	0.00024
STY2304	*rfbC*	STM2094	*rfbC*	dTDP-4-dehydrorhamnose 3,5-epimerase	Cell envelope	-3.48	0.00009
STY2346		STM2133		hypothetical protein	Unknown	-4.47	0.00035
STY2347		STM2134		hypothetical protein	Unknown	-2.40	0.00004
STY2378	*stcD, yehA*	STM2149	*stcD*	putative exported protein	Cell envelope	-2.85	0.00002
STY2403		STM2173		hypothetical protein	Unknown	-3.61	0.00001
STY2432		STM2198		putative transport protein (pseudogene)	Transport and binding proteins	-2.19	0.00004
STY2444		STM2208		putative membrane protein	Cell envelope	-4.39	0.00039
STY2469		STM2243		putative bacteriophage tail protein (pseudogene)	Unknown	-2.50	0.00003
STY2501		STM2274		putative transmembrane transpot protein (pseudogene)	Transport and binding proteins	-3.76	0.00016
STY2504		STM2275		putative transcriptional regulator (pseudogene)	Regulatory functions	-2.33	0.00002
STY2509		STM2280		putative transmembrane transport protein	Transport and binding proteins	-2.44	0.00026
STY2574		STM2344		putative sugar phosphotransferase component II A	Small molecule metabolism	-2.06	0.00010
STY2582	*hisM*	STM2352	*hisM*	histidine transport system permease	Transport and binding proteins	-2.61	0.00003
STY2607		STM2376		putative lipoprotein	Cell envelope	-2.15	0.00013

STY2608		STM2377		conserved hypothetical protein	Unknown	-2.76	0.00016
STY2621	*fadI*	STM2389	*yfcY*	putative 3-ketoacyl-CoA thiolase	Small molecule metabolism	-2.11	0.00009
STY2632	*pgtE, prtA*	STM2395	*pgtE*	outer membrane protease E, protease VII precursor	Cell envelope	-3.20	0.00038
STY2725	*purC*	STM2487	*purC*	phosphoribosylaminoimidazole-succinocarboxamide synthase	DNA/RNA replication, degradation, modification	-2.73	0.00020
STY2762	*sivH, sinH*	STM2517	*sinH*	putative intimin (pseudogene)	Pathogenesis	-2.32	0.00017
STY2820		STM2574		putative transmembrane transport protein	Transport and binding proteins	-2.57	0.00001
STY2860	*rplS*	STM2673	*rplS*	50S ribosomal subunit protein L19	Protein biosynthesis/degradation	-2.42	0.00008
STY2982	*ygbA*	STM2860	*ygbA*	conserved hypothetical protein	Unknown	-2.89	0.00010
STY2988	*sprA, hilC*	STM2867	*hilC*	possible AraC-family transcriptional regulator	Pathogenesis	-3.90	0.00002
STY2994	*prgI*	STM2873	*prgI*	pathogenicity 1 island effector protein	Pathogenesis	-3.42	0.00024
STY3004	*sipF*	STM2881	*iacP*	probable acyl carrier protein	Pathogenesis	-5.00	0.00003
STY3007	*sipC, sspC*	STM2884	*sipC*	pathogenicity island 1 effector protein	Pathogenesis	-2.01	0.00010
STY3010	*spaS*	STM2887	*spaS*	secretory protein (associated with virulence)	Pathogenesis	-2.05	0.00045
STY3047		STM2922		conserved hypothetical protein	Unknown	-3.08	0.00019
STY3167		STM3022		probable amino acid transport protein	Transport and binding proteins	-2.06	0.00043
STY3309		STM3138		hypothetical protein	Unknown	-2.43	0.00001
STY3331	*exbD*	STM3158	*exbD*	biopolymer transport ExbD protein	Transport and binding proteins	-6.04	0.00010
STY3332	*exbB*	STM3159	*exbB*	biopolymer transport ExbB protein	Transport and binding proteins	-3.53	0.00012
STY3340		STM3166.S		possible membrane transport protein	Transport and binding proteins	-2.58	0.00044
STY3456		STM3273	*yhbT*	conserved hypothetical protein	Unknown	-2.07	0.00027
STY3537		STM3357		possible transcriptional regulator	Regulatory functions	-4.71	0.00021
STY3605		STM3954	*yigG*	conserved hypothetical protein	Unknown	-3.03	0.00046
STY3862	*ompL*	STM4016	*ompL, yshA*	conserved hypothetical protein	Unknown	-2.78	0.00010
STY3871	*typA*	STM4009	*typA, bipA*	GTP-binding protein	Regulatory functions	-2.94	0.00049
STY4049	*trmH, spoU*	STM3743	*spoU*	tRNA (guanosine-2'-O)-methyltransferase	Protein biosynthesis/degradation	-2.53	0.00033
STY4072	*waaG, rfaG*	STM3722	*rfaG*	lipopolysaccharide core biosynthesis protein	Cell envelope	-3.25	0.00001
STY4077	*waaI, rfaI*	STM3718	*rfaI*	lipopolysaccharide 1,3-galactosyltransferase	Cell envelope	-6.82	0.00005
STY4081	*waaK, rfak*	STM3714	*rfaK*	lipopolysaccharide 1,2-N-acetylglucosaminetransferase	Cell envelope	-2.31	0.00007
STY4082	*waaL, rfaL, rfbT*	STM3713	*rfaL*	O-antigen ligase	Cell envelope	-2.26	0.00002
STY4106		STM3690		putative lipoprotein	Cell envelope	-4.77	0.00031

STY4264		STM3547.Sc		putative ribokinase	Unknown	-3.09	0.00022
STY4400	*metA*	STM4182	*metA*	homoserine O-succinyltransferase	Amino acid biosynthesis/degradation	-2.27	0.00015
STY4426	*malK*	STM4230	*malK*	maltose/maltodextrin transport ATP-binding protein	Transport and binding proteins	-2.01	0.00026
STY4453		STM4258	*siiB*	putative integral membrane protein	Pathogenesis	-3.55	0.00020
STY4456		STM4259	*siiC*	putative type-I secretion protein	Pathogenesis	-3.43	0.00002
STY4458		STM4261	*siiE*	large repetitive protein (pseudogene)	Pathogenesis	-2.35	0.00004
STY4582				possible exported protein	Pathogenesis	-2.89	0.00038
STY4679				putative membrane protein	Pathogenesis	-3.33	0.00001
STY4725	*rnr*	STM4368	*vacB*	ribonuclease R (RNase R)	DNA/RNA replication, degradation, modification	-3.84	0.00009
STY4728	*yjfJ*	STM4371	*yjfJ*	conserved hypothetical protein (pseudogene)	Unknown	-3.37	0.00004
STY4805		STM4467		arginine deiminase	Amino acid biosynthesis/degradation	-3.06	0.00024
STY4842				probable regulatory protein	Pathogenesis	-3.74	0.00000
STY4863	*trpS2*	STM4508	*trpS2*	probable tryptophanyl-tRNA synthetase	Protein biosynthesis/degradation	-2.85	0.00015
STY4881	*hsdS*	STM4524	*hsdS*	subunit S of type I restriction - modification system	DNA/RNA replication, degradation, modification	-2.51	0.00006
STY4899		STM4546	*yjjP*	putative membrane protein	Cell envelope	-2.96	0.00035
